# Volumetry may predict early renal function after nephron sparing surgery in solitary kidney patients

**DOI:** 10.1186/2193-1801-3-488

**Published:** 2014-08-29

**Authors:** Timur H Kuru, Jie Zhu, Ionel V Popeneciu, Nora S Rudhardt, Boris A Hadaschik, Dogu Teber, Matthias Roethke, Markus Hohenfellner, Martin Zeier, Sascha A Pahernik

**Affiliations:** Department of Urology, UniversityHospital Heidelberg, Im Neuenheimer Feld 110, 69120 Heidelberg, Germany; Department of Radiology, German Cancer Research Center, Heidelberg, Germany; Department of Nephrology, UniversityHospital, Heidelberg, Germany

**Keywords:** Nephron sparing surgery, Renal cell cancer, Renal function, Solitary kidney, Tumor volume

## Abstract

We investigate the impact of the residual kidney volume measured by tumor volumetry on preoperative imaging in predicting post-operative renal function. Nephron sparing surgery (NSS) in renal cell carcinoma (RCC) is the standard treatment for T1 kidney tumors. Resection of kidney tumors in solidary kidneys needs precise preoperative counseling of patients regarding post-operative renal function.

Patients planned for renal tumor surgery who underwent prior nephrectomy on the contralateral side were included. We identified 35 patients in our database that underwent NSS in solitary kidneys and met the inclusion criteria. Tumor volumetry was performed on computer tomography (CT) or magnetic resonance imaging (MRI) with the Medical Imaging Interaction Toolkit (MITK). Clinical and pathological data were assessed. Follow-up data included renal function over 3 years.

Mean age was 64 ± 8.1 years. Mean tumor volume on imaging was 27.5 ± 48.6 cc. Mean kidney volume was 195.2 ± 62.8 cc and mean residual kidney volume was 173.4 ± 65.3 cc. We found a correlation between renal function (MDRD) and residual kidney volume on imaging 1-week post-surgery (p = 0.038). Mid- and long-term renal function was not associated with residual kidney volume.

In conclusion, renal volumetry may predict early renal function after NSS.

## Introduction

Partial nephrectomy in renal cell carcinoma (RCC) should be the standard treatment for T1 kidney tumors in healthy patients (Ljungberg et al. 2010). The rationale for nephron sparing surgery (NSS) is possibly improved long-term survival of patients with preserved kidney function (Weight et al. 2010; Scosyrev et al. 2014). Several groups analyzed clinical parameters like pre-operative glomerular filtration rate (GFR) for prediction of renal function after NSS in recent years (Maehana et al. 2013; Mir et al. 2013). The use of imaging volumetry for prediction of renal function has also been evaluated in several studies (Patankar et al. 2013; Kunzel et al. 2013; Buethe et al. 2012). However, these studies were limited to patients with two kidneys who underwent NSS on one side. To our knowledge, no study investigated the impact of volumetry in CT or MRI in patients with solitary kidneys.

There are several studies which analyze the correlation of kidney volumetry and post-operative renal function in living kidney transplantation (Patankar et al. 2013; Kato et al. 2011).

Here, we investigated the impact of tumor volumetry on preoperative imaging in predicting post-operative early-, mid- and long-term renal function.

## Materials and methods

### Patient population

After receiving ethics committee approval and written informed consent, we identified 1538 patients in our prospective database, who were planned for renal tumor surgery at our institution between 2003 and 2011. Patients who underwent radical nephrectomy and patients with normal contralateral kidney were excluded from the study. Out of the remaining cohort, 130 patients underwent NSS in solitary kidneys. To eliminate compensating effects to kidney function, only patients were included which had undergone nephrectomy on the contralateral side due to renal cell cancer (at least 1 month prior NSS). Finally, we identified 35 patients who underwent NSS in solitary kidney for whom the glomerular filtration rate (GFR) was available preoperatively and postoperatively and preoperative imaging (MRI or CT) was digitally stored in our picture archiving and communication system (PACS). Regarding the cardiovascular status preoperatively, 15 patients suffered from arterial hypertension (AH), and two patients from AH and diabetes mellitus, 1 from AH and previous myocardial infarction.

All surgeons were experienced in performing kidney tumor resection. The procedure was performed in an in-house standardized technique. During surgery directly prior renal tumor resection, 20 mg furosemide was administered intra-venously.

All serum creatinine measurements were made at a single clinical reference laboratory, and GFR values were estimated using the “Modification of Diet in Renal Disease Study” (MDRD) (Levey et al. 1999) formula and the Chronic Kidney Disease Epidemiology Collaboration (CKD-EPI) (Levey et al. 2009) formula. Other clinical and pathologic features studied included age, sex, tumor volume, type and duration of ischemia. Patients were categorized regarding chronic kidney disease (CKD) post surgery (>6 months) in accordance to the K/DOQI guidelines (National Kidney Foundation 2002). All patients were included into a follow-up protocol with at least half-year visits including restaging with CT/MRI at our department.

### Imaging

**Figure 1:**
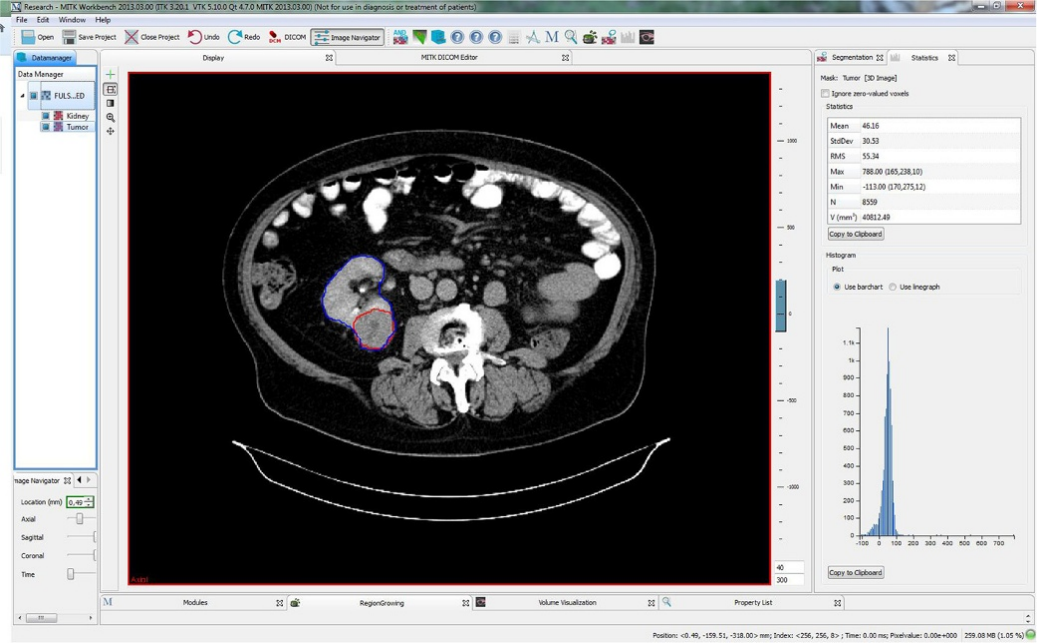
**Tumor (red circle) and kidney (blue circle) are contoured and the volume is calculated.**

All imaging was performed < 2 months prior surgery. MRI was performed in a 1.5 Tesla magnetic field. The standard abdominal MRI protocol was used, including a T2-weighted half-fourier acquisition turbo spin echo (HASTE) localizer sequence, an axial and coronal T1-weighted 3-dimensional gradient echo sequence (FLASH-3D), axial T2-weighted TSE sequence and fat saturated post-contrast axial and coronal T1-weighted sequences. Axial T1-weighted post-contrast images with 3 mm slice thickness were used for tumor measurement. Standard abdominal CT protocols were used for preoperative imaging. Volumetry was obtained from axial scans in the venous phase reconstructed at 3 mm intervals (MRI and CT) as illustrated in Figure 1. If MRI and CT imaging was available, CT imaging was used for tumor volume measurement.

### Volume analysis

Imaging data was evaluated using the Medical Imaging Interaction Toolkit (MITK) (Version 2013; DKFZ, Heidelberg, Germany, (http://www.MITK.org/Diffusion) (Fritzsche et al. 2012). Freehand drawing allows detailed discrimination of structures and was used to define the region of interest (ROI) in each image. In all instances, renal sinus fat, the collecting system, and non-enhancing cysts were excluded from the analysis. All volumetric analyses were performed blinded to the functional data.

Representative tumor regions were determined as regions of interest (ROI) on post-contrast T1-weigthed or venous phase images, respectively (Figure 1). In a second step, the kidney was contoured (ROI) in all images and the volume was calculated. Care was taken to include all of the tumor area in the ROI. Image processing estimated the total volume of renal parenchyma and tumor (Figure 1). Residual kidney volume expected post-treatment was assessed calculating the difference between total volume and tumor volume.

### Statistical analysis

Pearson’s correlation and Spearman’s correlation coefficient r_s_ were used to compare results of imaging volumes and clinical parameters. Normal distribution of the data was assessed using a Kolmogorov-Smirnov test. A p-value of 0.05 or less was considered as statistically significant. Multivariate analyses were done using linear regression models. All statistical analyses were two-tailed and performed with SAS V20.0 (SAS Institute, Cary, NC, USA).

## Results

Thirty-five (22 male, 13 female) patients were included into our analysis. The age ranged from 43 to 77 years (mean age 64 years). During follow-up, two patients underwent NSS for a second time due to local tumor recurrence three and five years after prior NSS on the same side. 24 (64.8%) tumors were localized on the right side, 13 (35.2%) on the left side. Cold ischemia was not applied due to relative small tumor volume. 14 (40%) patients underwent NSS without ischemia. Mean warm ischemia duration during NSS was 13 minutes (range: 8–38). Detailed patients’ data are listed in Table 1.

**Table 1:** **Patient data**

Number of patients (No. of tumors)	35 (37)
Age (y), (mean, range)	64 (43–77)
Sex	
Male	22
Female	13
Pathological tumor size (mean, median, range)	22.7 cc, 7.2 cc, (0.54 - 244.80)
Radiological tumor size (mean, median, range)	27.5 cc, 12.4 cc, (1.1 - 236.74)
Radiological kidney size (mean, median, range)	195.15 cc, 193.58 cc, (52.23 – 378.13)
No of. patients with Ischemia during NSS	
None	14
Warm (mean time, range)	23 (13 min, 8–38 min)

23 (62%) tumors were examined by MRI, 6 (16%) tumors by CT and 8 (22%) tumors were visualized by both MRI and CT prior to surgery. The median time between imaging and surgery was 1 ± 0.7 month. The mean tumor volume on imaging was 27.5 ± 48.6 cc. The mean pathological tumor volume was significantly lower (22.7 ± 43.4 cc, p < 0.001). The mean kidney volume was 195.15 ± 62.8 cc and the mean residual kidney volume was 173.43 ± 65.3 cc. Postoperatively one pneumothorax and one pulmonary embolism were observed. Long term follow up of renal function 12-, 24- and 36-months after surgery was available in 34, 28 and 20 patients, respectively. The mean GFR (MDRD) at baseline, 1-week, 6-months, 12-months, 24-months and 36-months post-surgery was 53.48 ± 16.38, 36.61 ± 22.77, 49.57 ± 15.53, 48.40 ± 20.99, 48.04 ± 18.06 and 50.72 ± 16.80, respectively. Results are listed in Table 2.

**Table 2:** **Longitudinal renal function**

	Baseline	1-week post surgery	6-months post surgery	12-months post surgery	24-months post surgery	36-months post surgery
Creatinine mg/dl (mean, range)	1.32 (0.76–2.72)	2.66 (0.69– 8.01)	1.39 (0.8– 2.57)	1.51 (0.7– 3.06)	1.49 (0.82– 3.14)	1.33 (0.74– 2.17)
GFR (MDRD) (mean, range)	53.48 (23.28– 90.58)	36.61 (6.8– 84.61)	49.57 (18.55– 87.19)	48.40 (15.95– 111.36)	48.04 (15.07– 86.67)	50.72 (23.28– 76.51)
GFR (CKD-EPI) (mean, range)	56.31 (22.64– 98.07)	38.03 (6.3– 89.74)	51.88 (18.54– 92.93)	50.11 (15.57– 101.39)	50.27 (14.85– 93.82)	52.89 (23.33– 79.50)

**Figure 2:**
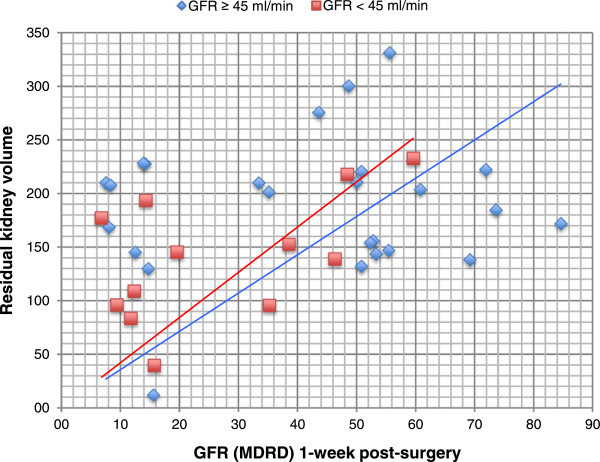
**Regression analyses of the renal function (MDRD) and the residual kidney volume on imaging 1-week post-surgery.** Patients with pre-operative GFR ≥ 45 and < 45 ml/min are shown in blue and red, respectively.

We found a significant correlation between the renal function (MDRD-EPI) and the residual kidney volume on imaging only 1-week post-surgery (p = 0.038). Regression analysis of these parameters was additionally performed (r_s_ = 0.75). The tumor volume showed no correlation to post-operative renal function (p = 0.1). We additional analyzed if a preoperative GFR ≥ or < 45 ml/min would influence the early postoperative function and found no significant differences (r_s_ = 0.71/0.69, Figure 2).

After NSS, 4 patients had CKD stage ≥ 4. In multivariate analyses neither residual kidney volume, time of ischemia, age, tumor volume on imaging nor preoperative GFR (MDRD) was a predictor for CKD stage ≥ 4 (Table 3).

**Table 3:** **Multivariate associations with CKD stage ≥ 4**

Parameter	Regression coefficient	Std. error	Sig.
Residual kidney volume	-.001	.001	.507
Time of ischemia	-.002	.004	.544
Age	.002	.007	.799
Tumor volume	.001	.001	.449
GRF pre-surgery	-.006	.003	.076

We analyzed the difference in predicting the early post-operative kidney function if tumor volume was measured in CT or MRI. There were no significant differences between both groups.

## Discussion

Our analysis demonstrates the feasibility of tumor volumetry on imaging (CT or MRI) for estimation of early post-operative renal function (p = 0.038). Therefore, the patient might be counseled by preoperative imaging about the expected postoperative renal function. Using volumetry Thompson et al. (2012) previously found a significant association between the preserved kidney volume in per cent (according to operation protocols) and the new-onset of chronic kidney disease stadium 4. In our study, there is a significant correlation between renal function after NSS and volumetry in the short-term, regardless if the pre-operative GFR was ≥ or < 45 ml/min. This correlation disappeared statistically over long-term (6, 12, 24, 36 months; p = 0.3, 0.09, 0.138, 0.2). Possible explanations may be the small number of patients in our cohort, which dilutes the results. On the other hand postoperative changes of the kidney, like hematoma or edema, may have played a role in the early postoperative renal function. As there is NO early imaging available, this study cannot further investigate these possible influences. Another explanation may be the resection of functional tissue during the tumor resection. During the procedure, the tumors were excised hardly at their capsule with a very small surgical margin around 2–3 mm. So the amount of excised functional kidney tissue is minimal and this point should also not influence the early renal function. On the other hand, compensation mechanisms of the kidney may play a role (Goldfarb et al. 2006). Kidney function over time may be influenced by other comorbidities of patients like arterial hypertension or diabetes mellitus, which were not stringently stored in our follow-up database. Due to the relatively low number of patients in our study, preoperative GFR showed only a trend in predicting severe chronic kidney disease in the multivariate analyses (p = 0.076). However, we were able to show that only a low number of patients (11%) developed higher grade CKD after NSS in solitary kidney. Known parameters (Chan et al. 2010) like time of ischemia, age or tumor volume did not show an influence in developing CKD ≥ stage 4. Regarding risk factors for renal failure in NSS, Maehana et al. (2013) reported that preoperative CKD stage 4 is a risk factor for temporary hemodialysis in the perioperative period.

Thompson et al. (2012), Simmons et al. (2012) and others reported a cut-off of 25 minutes warm ischemia time to effect renal function. The number of patients undergoing warm ischemia during NSS in our cohort is relatively high (23 of 35 patients, 65.7%) due to imperative indications and selection bias as a nationwide referral center for NSS. Nevertheless the mean time of warm ischemia is only 13 minutes, explaining the results why time of ischemia is not associated with higher stage CKD in our cohort.

We acknowledge that our multivariate analyses may be of weak statistical validity due to the relatively small number of patients. However, patients with solitary kidneys and malignant kidney tumors are relatively rare (Mues et al. 2012). For larger cohorts with more reliable statistical analyses, multicenter analysis may be the next step in generating more precise results in predicting kidney function in these patients. However, protocol variations might complicate such studies.

We found a significant difference of tumor volume between the radiological and pathological measurement (p < 0.001). As the radiological volume was higher, this difference may be most likely due to shrinkage during formalin fixation. We analyzed the influence of CT or MRI imaging used for volumetry in predicting post-operative renal function but found no significant differences. Thus, clinicians may easily replace one imaging modality by the other: in patients with iodine-allergy who cannot undergo contrast-enhanced CT imaging MRI can safely be used. On the other hand, patients who are not suitable to MRI examinations due to metal implants can be safely examined by CT imaging.

In the context of imaging and imaging scores, Buethe et al. (2012) investigated the use of the R.E.N.A.L. nephrometry score in predicting renal function after NSS and found no correlation. Bylund et al. (2012) found different results and were able to show correlation of R.E.N.A.L. and PADUA score with perioperative renal function impairment. In our understanding of RCC, the tumor itself has only marginal contribution to renal function due to its loss of original kidney cell function. This theory is supported by our results, that the kidney tumor volume itself did not show a correlation to the post-operative renal function (p = 0.1); only residual kidney volume did (p = 0.038). Similar results were reported by Mir et al. (2013) in 2013 in a non-homogenous cohort of bilateral and solitary kidney patients. In 2012, Jeon et al. (2012) already reported that preoperative GFR is an independent predictor of perioperative kidney failure in a heterogeneous patient-cohort undergoing radical or partial nephrectomy.

## Conclusion

Our data shows the impact of renal volumetry in predicting early renal function after NSS in solitary kidney patients. This effect is regardless of the image modality used and may play a role in counseling patient prior to surgery. The prediction of late GFR is not possible by tumor volumetry alone as it may be influenced by several comorbidities.

### Ethical standards

This study has been approved by the appropriate ethics committee (University Heidelberg) and has therefore been performed in accordance with the ethical standards laid down in the 1964 Declaration of Helsinki and its later amendments.
